# Two New Local Anæsthesia

**Published:** 1893-02

**Authors:** 


### Two New Local Anæsthetics.

The muriate of tropa-cocaine, or tropsin, is a substance obtained from the Java coca, and said by Professor Schweiger to have a local anæsthetic action resembling that of cocaine. It is weaker in action, but it causes anæsthesia more rapidly.

Eugenol-acetamide is recommended as a new anæsthetic. As crystallized from water it occurs in lustrous scales; from alcohol it occurs in fine needles, melting at 110° F.

This compound, when applied in the form of a fine powder, produces local anæsthesia without any caustic action, similar to cocaine. This anæsthetic effect, in connection with the strong antiseptic properties of eugenol-acetic acid, secures a place for this new amide in the treatment of wounds.—*N. Y. Med. Record.*

